# Rapid and effective fusion repair of severed digital nerves using neurorrhaphy and bioengineered solutions including polyethylene glycol: A case report

**DOI:** 10.3389/fncel.2022.1087961

**Published:** 2023-01-19

**Authors:** Stephen Lopez, George D. Bittner, Richard C. Treviño

**Affiliations:** ^1^Canton Plastic Surgery, Canton, OH, United States; ^2^Department of Neuroscience, University of Texas at Austin, Austin, TX, United States; ^3^WellSpan Medical Group, York, PA, United States

**Keywords:** peripheral nerve injury, polyethylene glycol, repair digital nerves, Wallerian degeneration, Semmes–Weinstein monofilament test, two point discrimination test

## Abstract

Peripheral nerve injuries (PNIs) that consist of simple nerve severance often result in severe motor impairment and permanent loss of function. Such patients face significant costs and pose major burdens to healthcare systems. Currently, the most promising surgical technique to achieve the best clinical outcome after such PNIs is immediate primary coaptation of severed nerve ends by microsutures (neurorrhaphy). However, recovery is often poor and delayed for many months due to Wallerian degeneration (WD) and slow (1–2 mm/day) axonal outgrowths from severed proximal axons that may not properly reinnervate denervated afferent/efferent targets that have atrophied. In contrast, recent pre-clinical studies using polyethylene glycol (PEG) to facilitate primary nerve repair have greatly improved the rate and extent of sensory and motor recovery and prevented much WD and muscle atrophy. That is, PEG-fused axons rapidly establish proximal–distal axoplasmic/axolemmal continuity, which do not undergo WD and maintain the structure and function of neuromuscular junction (NMJ). PEG-fused axons rapidly reinnervate denervated NMJs, thereby preventing muscle atrophy associated with monthslong denervation due to slowly regenerating axonal outgrowths. We now describe PEG-mediated fusion repair of a digital nerve in each of two patients presenting with a digital laceration resulting in total loss of sensation. The first patient’s tactile perception improved markedly at 3 days postoperatively (PO). Two-point discrimination improved from greater than 10 mm at initial presentation to 4 mm at 11-week PO, and the Semmes–Weinstein monofilament score improved from greater than 6.65 to 2.83 mm, a near-normal level. The second patient had severe PO edema and scar development requiring a hand compression glove and scar massage, which began improving at 11-week PO. The sensory function then improved for 4 months PO, with both two-point discrimination and Semmes–Weinstein scores approaching near-normal levels at the final follow-up. These case study data are consistent with data from animal models. All these data suggest that PEG-fusion technologies could produce a paradigm shift from the current clinical practice of waiting days to months to repair ablation PNIs with autografts, anucleated nerve allografts, or conduits in which the patient outcome is solely dependent upon axon regeneration over months or years.

## Introduction

Peripheral nerve injuries (PNIs) often produce impairments leading to long-term disabilities (Ruijs et al., 2005). Continued advances in surgical devices and procedures for nerve repair have had marginal success with patient satisfaction in motor recovery as low as 51.6% (Ruijs et al., 2005). Although limited data are available on their incidence, PNIs can lead to significant motor impairment and permanent loss of function, resulting in extraordinary costs and burdens to healthcare systems (Lundborg, 2005; Rosberg et al., 2005; Campbell, 2008; Brushart, 2011). Primary coaptations of severed proximal and distal nerve ends using microsutures (neurorrhaphy) currently produce the best outcomes clinically and in experimental animals, but recovery is still markedly delayed and poor due to Wallerian degeneration (WD) and slow nerve outgrowth at 1–2 mm per day often producing non-specific (if any) reinnervation of original sensory and motor target tissues (Mailander et al., 1989; al-Ghazal et al., 1994; Campbell, 2008; Brushart, 2011). This delayed reinnervation also limits recovery after neurorrhaphy due to muscle atrophy and loss of denervated end organs (Donaldson et al., 2002; Brushart, 2011). For example, an M3 motor grade (against gravity without resistance) and less than 15 mm two-point discrimination qualify as successful clinical outcomes.

Polyethylene glycol (PEG) has been used for its membrane fusogenic properties for many decades to make cell hybrids (Roos et al., 1983) and as an intravenous treatment for hemophiliacs (Johnson et al., 1971). Its fusogenic properties have successfully been applied to invertebrate giant axons and mammalian PNS and CNS axons *ex vivo* and *in vivo* (Bittner et al., 1986, 2012, 2016, 2022; Krause and Bittner, 1990; Lore et al., 1999; Donaldson et al., 2002). PEG-fusion repair of severed axons combined with primary nerve coaptation immediately and randomly joins (fuses/repairs) severed distal and proximal axonal ends to re-establish the structural and functional/electrophysiological continuity of 40–60% of severed myelinated axons across the lesion site. Successfully, PEG-fused axons do not undergo WD and exhibit minimal delamination or other changes in their myelin sheaths. PEG-fusion repair of singly cut PNIs immediately reinnervates many muscle fibers and sensory receptor endings. This rapid reinnervation is non-specific and produces immediate functional recovery of compound (nerve) action potentials and compound muscle action potentials but not immediate recovery of voluntary behaviors. Recovery of useful sensory/motor functions and voluntary behaviors takes 2–6 weeks and extensively involves many PNS and CNS plasticities (Mikesh et al., 2018a,b; Ghergherehchi et al., 2019; Bittner et al., 2022). The 40–60% of axons *not* PEG-fused undergo WD within 7 days and regenerating outgrowths arise from the severed ends of surviving proximal axons connected to their sensory cell bodies in dorsal root ganglia or motor cell bodies in the spinal ventral horn.

In this case study report, we apply the success of PEG-fusion previously demonstrated in animal models (Lore et al., 1999; Bittner et al., 2012, 2016, 2022; Bamba et al., 2016; Mikesh et al., 2018a,b) to present one of the first patient series in 2017–2018 in the United States to undergo FDA-approved PEG-fusion of human peripheral nerves.

## Case presentation

The trial was approved by the WellSpan Health Institutional Review Board. Trial registrations, www.clinicaltrials.gov NCT03236064. IND 118873 for PEG, were approved by the FDA. All participants provided written informed consent. The patients presented here are the first cases in our study series. Selection criteria were patients with a sharp, clean laceration and the ability for primary repair without tension within 24 h of the injury. Exclusion criteria were patients with traction injuries, inability to repair the nerve within 24 h, and the inability to primary repair without tension. We used two-point discrimination (2PD) and Semmes–Weinstein monofilament (SWM) tests (Bell-Krotoski et al., 1995; Silva et al., 2014). In brief, the normal range of digital 2PD is 2–8 mm, and 2.83–3.61 SWM sizes constitute a normal response.

All patients that met the inclusion criteria were given the opportunity to participate in our study. Patients were administered standard general anesthesia. The wounds were explored using loupe magnification. The nerve repairs were performed under the operating microscope. For our PEG-fusion protocol as published for the repair of singly cut rat sciatic nerves (Table 1), we irrigated the repair site with a calcium-free hypotonic solution (50% v/v normal saline in water) and used 9-0 nylon epineurial sutures to coapt the nerves. Methylene blue (1%, Faulding, Aguadilla, Puerto Rico) was then applied to the coapted site. PEG 50% w/w in sterile distilled water was subsequently added for 2 min to promote the fusion of the axolemmal leaflets. The lesion site was then irrigated with Lactated Ringer’s Injection solution (Hospira, Lake Forest, IL, USA) to completely remove the PEG and enhance axonal repair, that is, Lactated Ringer’s solution contains calcium, which helps seal any remaining holes in PEG-fused axons and seal the cut ends of any axons that did not successfully fuse (Table 1). The wounds were then closed with 4-0 nylon sutures, and the patients were splinted to facilitate recovery.

**TABLE 1:** Polyethylene glycol (PEG)-fusion protocol for repairing singly cut peripheral nerve injuries (PNIs).

Protocol Steps #1–5	Completely sever and trim nerve ends	Additive distress/toxicity: None Purpose: Prepare nerve ends for neurorrhaphy and PEG repair/fusion.
1. Priming Solution #1	Irrigation of surgical field with hypotonic Ca2+-free saline for 1–2 min	Additive distress/toxicity: None Purpose: Increase axoplasmic volume. Open cut axonal ends. Expel intracellular vesicles/organelles to enhance PEG-fusion.
2. Protection Solution #2	Administer 0.5% methylene blue (MB; an antioxidant) in distilled water for 1–2 min to opened cut ends	Additive distress/toxicity: None Purpose: Prevent formation of intracellular vesicles/organelles that interfere with PEG-fusion of cut ends and can seal-off each apposed cut end rather joining/fusing them.
3. Closely appose cut, opened, nerve ends	Perform neurorrhaphy	Additive distress/toxicity: None Purpose: Join nerve ends together for axonal sealing. Provide mechanical strength at each site of PEG-fusion.
4. PEG-fuse many axons Solution #3	Apply 50% w/w 3.35 kDa PEG in ddH_2_O for 1–2 min to the coaptation site	Additive distress/toxicity: None Purpose: Remove bound cell water to induce closely apposed, open, axonal membranes to non-specifically fuse.
5. Membrane repair Solution #4	Irrigation of coaptation site with isotonic Ca^2+^ -containing saline	Additive distress/toxicity: None Purpose: Induce vesicle formation to plug/seal any axolemmal holes after PEG-induced annealing of open cut axonal ends.

***Patient #1:*** A 35-year-old, right hand dominant, female patient lacerated her left index finger on a broken mirror at 1,500 h on 18 February 2017 and went to the emergency room secondary to a loss of sensation on the radial aspect of her left index finger. The laceration was on the radial side of the metacarpophalangeal flexion crease 7.5 cm from the tip of the finger. Her previous medical history included a storage pool defect, endometriosis, polycystic ovarian disease, and an adrenal mass. She smoked approximately half pack per day. The evaluation in the emergency room demonstrated no sensation present on the radial side of the index finger from the point of the laceration to the distal tip of the finger, with a 2PD response of greater than 10 mm and SWM response of greater than 6.65 mm. Noxious stimuli were applied by a 25-gauge needle and stabbed along with the radial aspect of the finger, including the pulp, without any perceived sensation. The sensation was intact and normal on the ulnar aspect of the index finger with 4 mm 2PD of 4 mm and 3.61 mm SWM.

Informed consent was obtained from patient #1 for PEG-fusion repair of the digital nerve, and the patient was then taken to the operating room at 0830 on 18 February 2017, i.e., within 24 h of injury. As observed under operating microscope high powered magnification, 50% of the radial digital nerve was lacerated, and the radial half had two clean-cut fascicles with a separation of less than 2 mm. Both the proximal and distal ends were easily identified and could be perfectly aligned. The epineurium was easily approximated, creating a direct fascicle-to-fascicle contact without bunching or axonal escape. The radial digital artery and flexor tendon sheath were intact. Post-surgery, the patient was placed in a protective dorsal blocking splint maintaining the index and middle fingers in the intrinsic plus position. The total time elapsed from the laceration to repair was 17.5 h.

**FIGURE 1:**
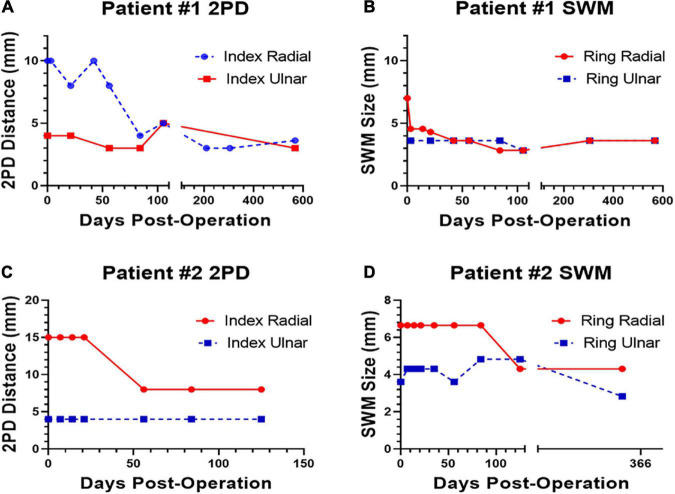
Polyethylene glycol (PEG)-fusion 2PD and Semmes–Weinstein monofilament (SWM) scores on the injured site (red, solid line) and on the uninjured site (blue, dotted line) plotted versus postoperatively (PO) days for Patient #1 **(A,B)** and Patient #2 **(C,D)**.

Patient #1 had subjective feeling in the tip of her index finger and responded to noxious stimuli at the time of discharge 1–2 h PO, and the results were notably better than her pre-operative evaluation. By 3 days PO, the largest type A fibers of the fingertip had some recordable sensation using SWM of 4.56 at the distal tip [The SWM examination demonstrated no sensation prior to intervention]. However, at 3-day PO, she initially complained of dull aching, some hypersensitivity, and lacked normal thermoreception, perceiving cold as wet. Her 2PD continued to improve over the next 70 days (Figure 1A). Her 2PD and SWM scores returned to near baseline levels by 80-day PO, which remained stable for 2 years PO (Figures 1A, B). At the last postoperative visit, the patient experienced cold stimuli as wet, suggesting that the smaller type Aδ and C fibers were altered, resulting in a lack of thermoreception.

***Patient #2:*** A 35-year-old male patient lacerated the palmar aspect of his left hand when cleaning a teapot, when it shattered (15 October 2017). Patient #2 reported copious bleeding that could not be controlled with a towel wrap. He called EMS and was brought into the emergency department with two forearm tourniquets in place. His past medical history was significant for anxiety and depression. His current medications were Paxil. The evaluation in the ER revealed a 3 cm transverse laceration at the base of the thenar eminence. Allen’s test was abnormally consistent with ulnar artery laceration. There was no sensation in the thumb, index, or middle fingers with 2PD greater than 10 mm. His 2PD on the small finger and ulnar aspect of the ring finger was 5 mm.

Informed consent was obtained from Patient #2 for PEG-fusion repair of the digital nerve, and he was taken to the operating room within 24 h of the injury. Exploration was performed under loupe magnification. The ulnar artery was completely lacerated and repaired with 8-0 nylon suture. The median nerve was completely lacerated within the carpal tunnel just proximal to its branching. Intraoperative nerve stimulation confirmed motor branch laceration and location. Accurate fasciclular alignment was achieved, and epineural repair was performed under the operating microscope with three 8-0 nylon sutures. Tension-free repair was noted with the wrist in 20^°^ of flexion and full finger extension; he was placed in a posterior splint maintaining wrist flexion of 20^°^ but allowed finger motion.

At 6 days PO, the 2PD of the thumb, index, and middle fingers was all greater than 10 mm. Thumb palmer abduction was weak. At 13-day PO, pinprick sensation was intact on the palmer aspect over the proximal phalanx of the index and middle fingers; 2PD was still greater than 10 mm at the finger pulps. At 21-day PO, Patient #2 demonstrated no improvement. At 35-day PO, his 2PD was 8 mm on the radial aspect of the ring finger and greater than 10 mm for the thumb, index, and middle fingers. His splint was discontinued and therapy (hand compression and glove and scar massage) commenced due to significant edema and scar development PO. At 8-week PO, Patient #2 reported painful stimuli from a splinter in one of his fingers and was able to perceive heat. His 2PD was 8 mm for the ring finger and greater than 8 mm for the thumb, index, and ring fingers (Figures 1C, D). At 4-month PO, Patient #2 reported continued improvement in sensation to affected digits, but his 2PD remained greater than 8 mm; his SWM was 6.65. At 11-month final follow-up, his SWM was 4.31 for the thumb, index, and middle fingers and 2.83 for the ring and small fingers (Figures 1C, D). There were no documented adverse events related to the PEG-fusion procedure through 11-month PO.

## Discussion

Patient #1 had a clean partial laceration to a pure sensory nerve. This minimally traumatic injury to an unifunctional nerve made PO assessments easier than if a mixed motor and sensory nerves were severed. This injury also allowed us to observe the outcomes of various sensory nerve fiber types, for example, mechanoreceptors, thermoreceptors, and nociceptors. At a rate of ∼1 mm per day, the proximal portion of the nerve should have taken 75 days to regenerate to begin to reinnervate terminal end organs. However, Patient #1 showed marked improvement in recovery time with recordable SWM and 2PD values as early as 0–20 days PO and a return to normal values by 70 days PO.

Patient #2 had a complete laceration of a mixed sensory and motor nerves. His motor recovery was more difficult to assess since he had weak abductor pollicis brevis function on presentation (indicating dual innervation). Patient #2 was lost to hospital follow-up for almost 4 months PO but was tested independently at 11 months by a nurse practitioner educated in proper SWM assessments. His 2PD was not collected, but his SWM was 4.31 at the thumb, index, and middle fingers and radial half of the ring finger. The ulnar half of the ring finger was 2.83. Subjectively, the patient was pleased with his result and continued to note improvement in sensation.

To our knowledge, there is only one other published international case study of PEG-fusion (Bamba et al., 2016) performed on four digital nerve transections in two patients, one boy (aged 16 years) and one girl (aged 17 years). All four nerve injuries were sharp complete transections and repaired without complications within 12 h post-injury. In both patients, gross sensation was observed within 1–3 weeks, comparable to our digital nerve laceration in which gross sensation was perceived almost immediately.

Human and experimental pre-clinical data on the repair of severance type PNIs by neurorrhaphy alone are remarkably similar, as human and experimental pre-clinical data are on the repair of severance type PNIs by neurorrhaphy with PEG-fusion technology. That is, the repair of severance type PNIs with neurorrhaphy alone produces (Campbell, 2008; Brushart, 2011; Bittner et al., 2012, 2016, 2022; Mikesh et al., 2018a,b):

(1) **Immediate loss of axonal integrity** so that action potentials are no longer conducted from the CNS to the periphery or from the periphery to the CNS across the injury site (s).
(2) **Immediate loss of sensation and muscle function** in the affected area, for example, the region of the limb previously innervated by the severed nerve distal to the injury site.
(3) **Irreversible WD** within 3–7 days of axons distal to the injury site.
(4) **Atrophy of muscle in the region of the limb previously innervated by the severed nerve distal to the injury site often** occurs before reinnervation.
(5) **Poor behavioral recovery after months/years**. Any sensation/functional recovery exclusively occurs using natural regeneration from surviving proximal axonal stumps that grow out at 1–2 mm/day. These phenomena are very similar to those observed for humans in clinical settings.

In contrast to (**1**)–(**5**) listed above, experimental animals having cuts repaired by neurorrhaphy and PEG-fusion exhibit (Bittner et al., 2012, 2016, 2022; Mikesh et al., 2018a,b):

1*) **Axonal integrity rapidly (within minutes) restored** as assessed by (**a**) conduction of extracellularly recorded compound action potentials (CAPs) intracellular dye diffusion in both directions across the lesion site(s), (**b**) conduction of compound muscle action potentials (CMAPs) to muscle groups distal to the lesion, **(c)** retrograde fast axonal transport restored 0–2 days postoperatively (PO) following PEG-fused single cut lesions or 14–17 days PO following PEG-fused ablation lesions.
2*) **Neuromuscular junctions (NMJs) continuously maintained,** as measured by CMAPs confocal immunohistochemistry, TEM, counts of innervated muscle fibers, and evoked muscle twitches.
3*) **No WD of distal segments of many host or donor graft axons**, as assessed by photon microscopy and TEM of cross and longitudinal sections.
4*) **Reduced atrophy (compared with controls) of muscle fibers**, as assessed by TEM.
5*) **Functional/behavioral recovery restored within days to weeks to levels that can approach that of unoperated animals,** as measured by the Sciatic Functional Index (**SFI**), a commonly used behavioral measure primarily determined by fine sensory and motor control of distal muscle masses responsible for toe spread and foot placement.

The sensory aspects of phenomena in experimental animals are similar to those reported for our two patients and those of Bamba et al. (2016).

One potential drawback to PEG-fusion is its lack of initial specificity in mixed nerve repairs. That is, motor axons can be fused with sensory. While this efferent/afferent mismatch is possible, the degree could be limited by two mechanisms. The first would be accurate fascicle alignment. Knowledge of peripheral nerve topography has revealed motor/sensory segregation. While it is not possible to 100% accurately align the fascicles, the use of the operating microscope and nerve surface landmarks should achieve alignment sufficient to allow motor/sensory nerve groups enough overlap to achieve target success. The other mechanism is 40–60% of axons in a nerve are typically repaired by PEG-fusion (Mikesh et al., 2018a,b). The axons that do not fuse can regenerate in the usual fashion and produce improvement in sensory perceptions, as observed in both patients, that is, Patient #2 demonstrated marked improvement in sensation at 11-month PO, and Patient #1 showed rapid improvement in SWM values but slow improvement in 2PD values.

## Conclusion

Our clinical data suggest that PEG-fusion repairs of PNIs have the potential for much improved clinical outcomes when compared with traditional coaptation repairs, especially in proximal mixed motor/sensory nerves. The adoption of PEG-fusion to treat PNIs would constitute a paradigm shift not only in functional outcomes but also in clinical care. While there is no consensus on the post-injury timing for addressing transection PNIs (Pan et al., 2020), current clinical practices with PNIs are often to wait days or weeks following the PNI before repairing (Campbell, 2008). This practice of waiting to address the PNI is not a clinical necessity and outcomes with PNIs addressed at different times within weeks of the injury do not differ (Jones et al., 2018). Thus, while delayed repair (days–weeks post-PNI) can be helpful for more clearly assessing the extent of nerve damage and more favorable scheduling of procedures, it is not a necessary clinical practice—particularly for those patients with complete lesions where the need for surgery is already certain, which is the initial target patient population for the PEG-fusion technology. While outcomes with current technologies do not require early intervention, the benefits of immediate reinnervation through PEG-fused axons in patients would warrant a shift in clinical practices toward treatment within 72 h or less (determined by experiments on experimental animals). Most other clinical practices associated with the treatment of PNIs, such as controlling neuropathic pain, would likely be the same (Dworkin et al., 2003).

## Data availability statement

The original contributions presented in this study are included in the article/supplementary material, further inquiries can be directed to the corresponding author.

## Ethics statement

The studies involving human participants were reviewed and approved by WellSpan Health Institutional Review Board. Trial registrations, www.clinicaltrials.gov NCT03236064. IND 118873 for PEG was approved by the FDA. The patients/participants provided their written informed consent to participate in this study. Written informed consent was obtained from the individual(s) for the publication of any potentially identifiable images or data included in this article.

## Author contributions

SL and RT performed the surgeries and oversaw patient follow-up. All authors helped design the study, analyzed data, wrote and edited various drafts of the manuscript, and approved the submitted version.
